# Remodeling cancer stemness by collagen/fibronectin *via* the AKT and CDC42 signaling pathway crosstalk in glioma

**DOI:** 10.7150/thno.50613

**Published:** 2021-01-01

**Authors:** Chuanhong Zhong, Bei Tao, Fangli Tang, Xiaobo Yang, Tangming Peng, Jian You, Kaiguo Xia, Xiangguo Xia, Ligang Chen, Lilei Peng

**Affiliations:** 1 Department of Neurosurgery, Affiliated Hospital of Southwest Medical University, Luzhou China.; 2 Sichuan Clinical Research Center for Neurosurgery, Luzhou, China.; 3 Academician (Expert) Workstation of Sichuan Province, Luzhou China.; 4 Neurological Disease and Brain Function Laboratory, Luzhou, China.; 5 Department of Rheumatism, Affiliated Hospital of Southwest Medical University, Luzhou, China.; 6 The Second People's Hospital of Yibin, Yibin, China.

**Keywords:** collagen, fibronectin, glioma, PI3K/AKT, CDC42

## Abstract

Cancer development is a complex set of proliferative progression, which arises in most cases via multistep pathways associated with various factors, including the tumor microenvironment and extracellular matrix. However, the underlying mechanisms of cancer development remain unclear and this study aimed to explore the role of extracellular matrix in glioma progression.

**Methods:** The expression of type I collagen and fibronectin in tumor tissues from glioma patients was examined by immunofluorescence staining. The correlations between collagen/fibronectin and glioma progression were then analyzed. A 3D collagen/fibronectin cultured system was established for tumor cells culture *in vitro*. Quantitative, real-time PCR and western blot were used to detect PI3K/ATK and CDC42 signals associated proteins expression in glioma. We used *in vitro* Cell Counting Kit-8, colony formation, and tumorigenesis assays to investigate the function of PI3K/AKT and CDC42 signals associated proteins. A xenograft glioma mice model was also used to study the anticancer effects of integrin inhibitor *in vivo*.

**Results:** Our study demonstrated that type I collagen and fibronectin collaborate to regulate glioma cell stemness and tumor growth. In a 3D collagen/fibronectin culture model, glioma cells acquired tumorigenic potential and revealed strengthened proliferative characteristics. More significantly, collagen/fibronectin could facilitate the activation of PI3K/AKT/SOX2 and CDC42/YAP-1/NUPR1/Nestin signaling pathways *via* integrin αvβ3, eliciting sustained tumor growth and cancer relapse. Combination of the integrin signaling pathway inhibitor and the chemotherapeutic agent efficiently suppressed glioma cell proliferation and tumorigenic ability.

**Conclusion:** We demonstrated that type I collagen and fibronectin could collaborate to promote glioma progression through PI3K/AKT/SOX2 and CDC42/YAP-1/NUPR1/Nestin signaling pathways. Blockade of the upstream molecular integrin αvβ3 revealed improved outcome in glioma therapy, which provide new insights for eradicating tumors and reducing glioma cancer relapse.

## Introduction

Glioma is the most common brain malignant disease with a high incidence worldwide 1, 2. Despite maximal surgical resection followed by adjuvant radiation and chemotherapy, the disease still has a high potential for recurrence due to residual tumorigenic cells and cancer stem cells (CSCs) 3. The median survival of glioma patients remains 12-16 months, and the 5-year survival rate is less than 10% 3. Several crucial reasons are believed to be associated with the poor outcome in glioma patients, including the pro-tumor microenvironment and the sustained tumor growth induced by the cancer stem cells 4-8. There is a critical need to elucidate the underlying mechanisms of glioma progression and develop novel therapeutic strategies to target tumor progression and decrease recurrence rates.

Glioma stem cells are considered crucial drivers for frequent relapse and persistent para-cancerous tissue invasion during glioma therapy 7-10. Cancer stem cells, also named tumor-initiating cells or tumor repopulating cells, are defined by specific cell surface markers and their capacity for self-renewal and tumorigenesis 11, 12. Cancer stem cells appear to be inherently resistant to chemotherapeutic agents and are involved in tumor relapse and distant metastasis, resulting in poor outcomes after standard clinic therapies 12. However, the identity of cancer stem cells remains controversial with the outstanding question of whether CSCs are intrinsic sub-population in tumor cells defined by their plasticity, which could be altered by microenvironmental cues 13. Increasing evidence has highlighted the impact of the tumor microenvironment on CSC functionality and spatiotemporal regulation, resulting in cancer heterogeneity 14, 15. Further, recent studies revealed that opportune culture conditions, such as biomaterial 3D fibrin or collagen culture system, efficiently strengthen stem-like phenotypes and facilitate the tumorigenesis/proliferation capability of cancer cells, suggesting a dynamic bidirectional conversion between CSCs and bulk cancer cells highly dependent on the microenvironment 13, 16. The extracellular matrix and its sequestered growth factors are a fundamental component of the tumor microenvironment.

The extracellular matrix comprises proteins, glycoproteins, polysaccharides, and cytokines, and multiple types of stromal cells 17, 18. Mounting evidence has demonstrated that the extracellular matrix is essential for cancer cell stemness maintenance and tumor development 19, 20. For example, the distribution of several types of collagens in the extracellular matrix is strictly correlated with the prognosis of bladder cancer patients 21-26. Similarly, the Wnt/β-Catenin pathway induces the stem-like phenotype in ovarian cancer cells and is activated by the extracellular matrix complex 27. Several extracellular matrix-associated tumor receptors, such as integrin β3, serve as markers to identify cancer stem cells, suggesting that the functional states and phenotypes of cancer stem cells might be a consequence of microenvironmental cues and the extracellular matrix is central to cancer stem cell heterogeneity or tumor signaling ligand activation 22, 28-30. However, there is a lack of evidence explaining how tumor cells are triggered into stem-like phenotypes by the extracellular matrix. Thus, successful anticancer stem cell therapies have yet to be developed.

In this study, we provided evidence to suggest that glioma cells are plastic and the type I collagen/fibronectin (FN) in tumor extracellular matrix could strengthen the tumorigenic potential and proliferative characteristics of glioma cells, leading to the malignant progression of glioma. Further, we described an improved biomaterial 3D collagen/FN gel model for glioma cell culture, providing an ideal culture system to induce cancer cells with a stem-like phenotype and a model to simulate the *in vivo* cancer stem cell “niches” during tumor progression. More importantly, we identified that collagen/FN stimulated glioma progression through integrin αvβ3-induced PI3K/AKT/SOX2 and CDC42/F-actin/YAP-1/Nupr1/Nestin double signal activation simultaneously, expounding the underlying mechanism of the extracellular matrix-induced tumor signaling network. Significantly, we employed the combination of oral integrin αvβ3 inhibitor SB273005 and chemotherapeutic temozolomide and demonstrated efficient suppression of glioma progression, providing an innovative therapeutic strategy for clinical glioma therapy.

## Methods

### Materials

Collagenase, fibronectin, and NaOH were purchased from Sigma-Aldrich (USA). The inhibitor of PI3K (LY294002), 10×PBS was acquired from Invitrogen (MA, USA). The inhibitors of integrin αvβ3 (SB273005) and AKT (MK-2206) were obtained from Top science (Shanghai, China). Temozolomide was purchased from Selleck Chem (NJ, USA).

### Cell culture system and reagents

For the conventional 2D cell culture, 293T, LN229, and T98G cells were cultured in regular tissue culture dishes with Dulbecco's Modified Eagle's Medium (DMEM) supplemented with 10% fetal bovine serum (FBS) (Hyclone, MA, USA), 2 mM L-glutamine, penicillin (100 U/mL, Thermo, MA, USA), streptomycin (100 μg/mL, Thermo, USA). 3D fibrin gel culture was conducted according to a previously described method 13.

Briefly, human fibrinogen (Sigma, MA, USA) was dissolved at 2 μg/μL in dd H_2_O. Human fibrinogen and cell solution were 1:1 mixed to produce 1 μg/μL fibrinogen/cell solution. 50μLfibrinogen/cell solution (including 1000 cells) were seeded into each well of 96-well plate and mixed well with 1μL thrombin (0.1 U/μL, Searun Holdings Company), resulting in the 3D fibrin gel. After 2 h, DMEM supplemented with 10% FBS, 2 mM L-glutamine, 100 U/mL penicillin, and 100 μg/mL streptomycin was added to the 3D fibrin gel plate.

The 3D collagen gel culture was conducted according to a previously described method. Collagen I (Corning, 354236, NJ, USA) was neutralized with 100 mM HEPES, 0.1 M NaOH and 10 × PBS, pH 7.4, and dissolved in DMEM at 2 μg/μL. Neutralized collagen and cell solution were mixed 1:1 to produce 1 μg/μL collagen/cell solution. 250 collagen /cell solution (including 8000 cells) were seeded into each well of the non-tissue 24-well plate. After polymerization at room temperature for 20 min and at 37 ºC for 1 h, DMEM supplemented with 10% FBS, 2 mM L-glutamine, 100 U/mL penicillin, and 100 μg/mL streptomycin was added to the 3D collagen gel plate. 3D collagen/FN gel is improved on conventional 3D collagen gel culture. After neutralizing the collagen solution, the gel solution was added to a certain concentration FN and resuspended with cells. Next, 3D collagen gel/FN culture was conducted according to the 3D collagen gel method.

### Clinical specimens

Primary glioma tumor tissues were sterilely acquired after the surgery at the Affiliated Hospital of Southwest Medical University. All patients were newly diagnosed glioma patients who had not received any prior therapies, agreed to participate in this research, and provided written informed consent. Sample collection and processing were carried out according to the Declaration of Helsinki. This study was approved by the Ethics Committee of the Affiliated Hospital of Southwest Medical University. All clinical specimens were classified based on the AJCC staging system.

### Lentiviral transduction

Lentivirus was obtained from SyngenTech (Beijing, China). The viruses were mixed with polybrene to a final concentration of 8 μg mL^-1^ before infection of LN229 or T98G cells. The following sequences of human SOX2 shRNA were used: CCGGCAGCTCGCAGACCTACATGAACTCGAGTTCATGTAGGTCTGCGAGCTGTTTTTG. The sequences of human NESTIN shRNA were as follows: CCGGAGAGGCTGTAGGCCAACTTAACTCGAGTTAAGTTGGCCTACAGCCTCTTTTTTTG. Sequences of human CDC42 cDNA were as in NM_044472.3. Cells with stable knockdown of SOX2, Nestin or overexpression of CDC42 were sorted with FACS (BD Biosciences, MA, USA).

For stable knock-out of ITGB3, 2×10^5^ LN229 or T98G cancer cells were seeded in a 6-well plate. After 8 h, cells were transfected with 5 μg of a px459 vector expressing sgRNAs targeting ITGB3(sg1: CCTCGCGTGGTACAGATGTT, sg2: ACCTCGCGTGGTACAGATGT) using Lipofectamine 2000 (Thermo Fisher Scientific Inc, MA, US) according to manufacturer's instructions. After 48 h, cells were treated with puromycin (1 μg/mL, Thermo Fisher Scientific Inc, MA, US) for 72 h. Growing isolated clones were cultured in 96 well plates (Thermo Fisher Scientific Inc, MA, US). Each clone was analyzed for ITGB3 expression by Western blotting.

### Cell proliferation and colony formation assays

LN229 or T98G cell proliferation was assessed by Cell Counting Kit-8 (CCK-8, Solarbio, Beijing, China). Briefly, glioma cells were seeded in 96-well plates (5000 per well) in triplicate. Cell proliferation was determined at 0, 24, 48, and 72 h according to the manufacturer's protocol and absorbance was quantified at 450 nm by Gen5 (Agilent Technologies, MA, USA).

For the colony formation assay, LN229 or T98G cells were seeded in 6-well plates at 500 or 1000 per well and cultured in DMEM with 10% FBS for 24 h in a humidified incubator. After 2 weeks, colonies were fixed with methanol at room temperature for 10 min and stained with crystal violet (Solarbio, Beijing, China) at room temperature for 5 min. The cells were washed with PBS three times. Visible colonies were counted.

### 3D colony size assay

A total of 5000 LN229 or T98G cells were seeded in collagen/FN gel and cultured for five days. Colony images were taken at different time points by a CCD camera under the white light of a microscope (Olympus, Tokyo, Japan) (200 ×). The colony size was analyzed by Image J software.

### Tumor growth and tumorigenesis assay

The animal protocol of this study was approved by the Institutional Animal Care and Use Committee (IACUC) of Southwest Medical University (Luzhou, China). NSG mice were obtained from Beijing HFK Inc., China. LN229 or T98G cells were injected into NSG mice (10^5^ cells for each tumor) subcutaneously for the tumorigenesis assay for 3 weeks according to published methods. To develope the collagen and FN environment *in vivo*, collagen /FN gel (50μg collagen and 50 ng FN in 50 μL PBS/tumor) was injected into the tumor sites on day 7 and 14. After 10 days, tumor size was measured at different time points, and the survival rates were recorded.

### Immunofluorescence analysis

Tumor tissues and the 3D gel were fixed with 4% paraformaldehyde for 72 h, sectioned, and subjected to sodium citrate antigen retrieval. LN229 or T98G cells were seeded on glass cover-slips in 6-well plates. The tissue and 3D gel sections and glass cover-slips with cells were fixed with 3% PFA/PBS for 20 min at 4 °C). The cells on glass cover slips were permeabilized with 0.1% Triton X-100/PBS for 10 min at room temperature. After washing with PBS three times, tissue sections and glass coverslips with cells were blocked with 5% goat serum in PBS for 30 min. Next, the sections and glass cover-slips were incubated with primary anti-p-PI3K^Tyr607^ antibody (ab182651, Abcam, Cambridge, UK) and anti-p-AKT1^S473^ antibody (ab8932, Abcam, Cambridge, UK) for overnight at 4 °C, followed by incubation with goat anti-rabbit Alexa Fluor® 488 or donkey anti-rabbit Alexa Fluor® 594 secondary antibodies (Abcam, Cambridge, UK). Nuclei were stained with DAPI (Solarbio, Beijing, China) at room temperature for 5 min. Subsequently, photographs were acquired by a laser scanning confocal microscope (Olympus imaging Inc., Tokyo, Japan). The relative protein expression was analyzed by the fluorescence intensity. The mean fluorescence intensity (MFI) of a field (at least 50 tumor cells in each field) was calculated by image J software. A mean of 20 fields MFI was calculated as the MFI of the sample. A total of 10 samples in each group were used for the significant difference analysis.

### Dual-luciferase reporter assay

The NUPR1 promoter sequence was inserted into the pGL4.11 vector containing the luciferase gene. LN229 or T98G cells were co-transfected with Renilla vectors and pGL4.11-NUPR1 using FuGENE HD Transfection Reagent (Roche, NJ, USA) for 6 h. Next, LN229 or T98G cells were transfected with pCMV-YAP1 (Origene, CA, USA) and pCMV-TEAD4 (Origene, CA, USA) vectors using FuGENE HD transfection reagent (Roche, NJ, USA) for 48 h. Luciferase activities were measured using the dual-luciferase assay kit (Beyotime, Beijing, China). The activity of luciferase was normalized to that of Renilla luciferase.

### Chip Assay

The CHIP Kit (ab500, Abcam, Cambridge, UK) was used according to the manual instructions. The LN229 or T98G cells cultured in 10 cm dishes were crosslinked with 1% formaldehyde in PBS and plates were incubated on a rotator for 10 min at 25 °C. Subsequently, formaldehyde was removed and crosslinking quenched by incubation with 125 mM glycine in PBS for 5 min at 25 °C. Following solution removal, plates were chilled on ice and the cells lysed by adding 2 mL of cold lysis buffer with a protease inhibitor (04693116001, Roche, USA). The chromatin was fragmented to 200-500 bp with a Misonix S3000 Sonicator (Farmingdale, USA) at 4 °C. After centrifugation at 10,000 × g for 5 min at 4°C, chromatin supernatants were diluted with cold IP dilution buffer. The human YAP1 antibody (1 µg, ab52771, Abcam, Cambridge, UK) or human TEAD4 antibody (1 µg, ab58310, Abcam, Cambridge, UK) was added to chromatin and the mixture was incubated at 4 °C overnight. Dynabeads were added to the chromatin/antibody mixture and incubated for additional 4 h at 4 °C. Beads were washed with the wash buffer and samples were eluted with 250 µl elution buffer. The eluted samples were treated with 0.2 M NaCl and 1 mg/mL Protease K at 65 °C overnight. Chip samples were purified with phenol/chloroform and precipitated with cold ethanol and glycogen.

qPCR analysis was performed on the CFX96 Touch Real-Time PCR system (Bio-Rad, MA, USA) using a SYBR Premix (Bio-Rad, MA, USA) according to the manufacturer's instructions. The primer sequences used were as follows: NUPR1-F: 5ʹ-GATCAGCCTGTCCAACATGGTGAAAC-3ʹ; NUPR1-R: 5ʹ-TTTGAAATGGAGTCTCTGTC-3ʹ.

### Western blotting

Cell lysates were separated on 10% sodium dodecyl sulfate-polyacrylamide gels (SDS-PAGE). Western blotting was performed as described in our previous study 31. The antibodies used were against human proteins ITGB3 (#4702,CST,USA), integrin alpha V beta 3 (ab78289, Abcam, Cambridge, UK), CDC42 (#2466,CST, NJ, USA), F-actin (#206,Abcam, Cambridge, UK) , NUPR1 (#6028, Abcam, Cambridge, UK) , YAP1 (#52771, Abcam, Cambridge, UK) , and p-YAP1-S127 (#76252, Abcam, Cambridge, UK). β-actin (#179467, Abcam, Cambridge, UK) was used as the protein loading control.

### Mass spectrometry and co-immunoprecipitation

LN229 cells were harvested, lysed, and immunoprecipitated with anti-YAP1 (MAB8094, RD, USA) or IgG conjugated to protein G agarose (11243233001, Roche, USA) at 4 ºC overnight. Then, the immunoprecipitates were separated on SDS-PAGE gels. After coomassie blue staining, the protein bands were collected for mass spectrometry analysis.

The monolayer cells or 3D cells were lysed in the IP lysis buffer (pH 7.4, 25 mM Tris, 150 mM NaCl, 1 mM EDTA, 1% NP40, 5% glycerol, P0013, Beyotime, China). The proteins were immuno-precipitated with anti-YAP1 (MAB8094, RD, USA) or IgG conjugated to protein G agarose (11243233001, Roche, USA) at 4 ºC overnight. Subsequently, the immuno-precipitates were used for Western blotting analysis following the procedure described in our previous study 31. The antibodies used were against human TEAD4 (ab151274, Abcam, Cambridge, UK) and YAP1 (ab52771, Abcam, Cambridge, UK). β-actin (ab179467, Abcam, Cambridge, UK) was used as the protein loading control.

### Statistical analysis

Each experiment was conducted at least three times independently. Results were presented as the mean ± SEM and statistical significance was analyzed using GraphPad 7.0 software. Statistical significance between groups was calculated by Student's t-test for two groups or by one-way ANOVA for more than two groups. The survival rates were determined by Kaplan-Meier survival analysis (*p < 0.05; **p < 0.01; ns, no significant difference).

## Results

### 3D collagen I/FN gel facilitates glioma cell proliferation and tumor growth

**Figure 1:**
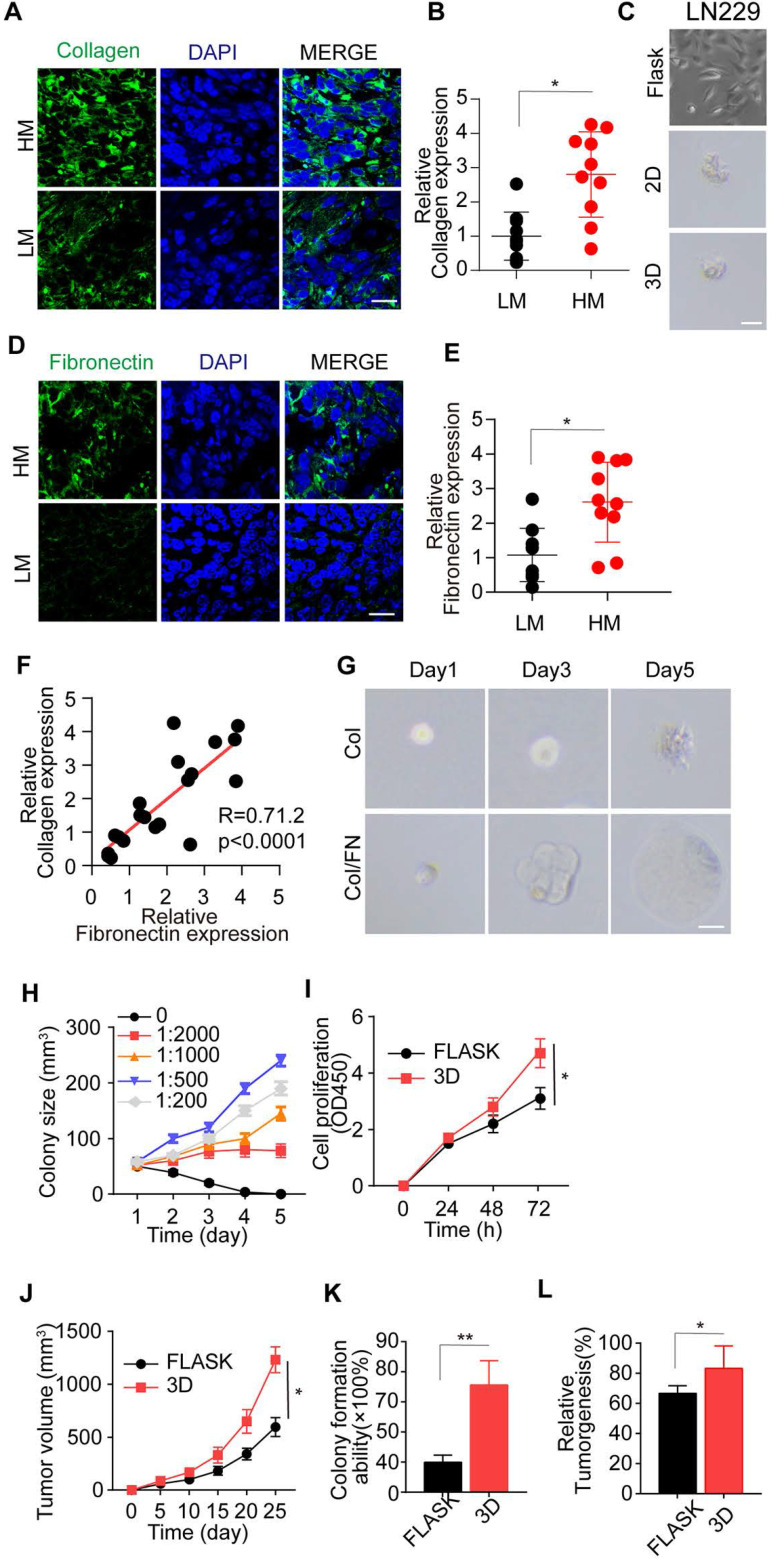
** The optimal 3D collagen I/FN gel facilitates glioma cell proliferation and tumor growth.** (A) Immunofluorescence staining of type I collagen in high-grade malignant (HM, WHO 3~4) or low-grade malignant (LM, WHO 1~2) gliomas. Scale bar represents 20 µm. (B) Relative expression of type I collagen in glioma tissues from high-grade malignant (HM, WHO 3~4) or low-grade malignant (LM, WHO 1~2) gliomas by immunofluorescence analysis, n=10. (C) Representative photographs of LN229 cells cultured in flasks, 2D collagen, or 3D collagen gel for 72 h. Scale bar represents 20 µm. (D) Immunofluorescence staining of FN in HM and LM glioma tissues. Scale bar represents 20 µm. (E) Relative expression of FN in HM and LM glioma tissues by immunofluorescence analysis, n=10. (F) Correlation study between type I collagen and FN in glioma tissues from patients. (G) Representative photographs of LN229 cells cultured in 3D collagen and LN229 cells cultured in 3D collagen with FN (FN: collagen, 1:500) for 24, 48, 72 h. Scale bar represents 20 µm. (H) LN229 cells cultured in 3D collagen with FN. Quantification of colony size (N = 50). (I) Viability of LN229 cells cultured in a flask and in 3D Collagen/FN (collected after 3 days culture) was measured using the CCK-8 assay for 0, 24, 48 and 72 h (J) Tumor volume of xenografts in NSG mice (n=10) injected with 1×10^6^ LN229 cells cultured in a flask or 3D Collagen/FN. (K) Colony formation analysis of LN229 cells cultured in a flask or 3D Collagen/FN environment for 3 days. (L) Tumorigenesis of LN229 cells cultured in a flask or 3D Collagen/FN for 3 days (n=6). Mean ± SEM, n.s, no significant difference, *p < 0.05, **p < 0.01.

Studies in a range of mammalian organ systems have demonstrated that ECM components revert tumor cells into functional stem-like tumor cells, resulting in sustained tumor growth 21, 22. Type I collagen is the major structural protein of the extracellular matrix in the tumor microenvironment, tightly associated with tumor progression 32, 33. Herein, we examined the expression of type I collagen glioma patients' tumor tissues and observed its increased expression in high-grade malignant glioma tissues compared to low-grade gliomas (Figure 1A-B). To further investigate type I collagen-induced tumor progression, we added collagen (0.1-10 μg/mL) to the culture medium; however, the treatment did not induce glioma cell proliferation (Figure S1A and B). Similarly, the 2D or 3D collagen culture also elicited a high percentage of cell death and failed to sustain the long-term viability of glioma cells (Figure 1C and Figure S1C-E). These results indicated that other elements in ECM might participate in the collagen-induced tumor progression and glioma malignancy. FN, a high-molecular weight (~440 kDa) soluble glycoprotein of the extracellular matrix, could enlist collagen or fibrin and bind to membrane-spanning receptor integrins, leading to ECM-induced signal transduction 17, 34, 35. Therefore, we examined FN expression in tumor tissues from glioma patients and observed increased FN expression in high malignant gliomas (Figure 1D-E). The elevated FN expression was highly correlated to collagen expression (Figure 1F), indicating a potential link between FN and collagen in inducing glioma progression.

Thus, we established a 3D collagen/FN model for glioma cell culture. Remarkably, the addition of FN to the 3D collagen model efficiently facilitated glioma cell proliferation and spheroid colony formation (Figure 1G and Figure S1F), whereas the addition of FN alone in the culture medium could not facilitate tumor cell proliferation (Figure S1G and H). This result suggested that FN, together with collagen, transduced the pro-survival signals to glioma cells in the 3D culture system or tumor tissues and did not directly regulate glioma cell proliferation. Furthermore, at the 1:500 concentration of FN and collagen, the glioma cell proliferation was markedly increased in the solid 3D culture model, indicating it to be the optimal concentration (Figure 1H and Figure S1I).

To further investigate the proliferative and tumorigenic potential induced by collagen/FN, glioma cells were cultured in the 3D collagen/FN system for 3 days and collected for cell proliferation and tumorigenesis analysis. As shown in Figure 1I-J and Figure S1J-K, the 3D collagen/FN system remarkably facilitated glioma cell proliferation *in vitro* and *in vivo*. The glioma cells isolated from the collagen/FN system revealed the enhanced capability of colony formation/growth (Figure 1K and Figure S1L) and tumorigenesis (Figure 1L and Figure S1M). Together, those results indicated that the collagen and FN in ECM facilitated glioma cell proliferation and tumorigenesis.

### 3D collagen I/FN gel induces tumor progression via integrin αvβ3

**Figure 2:**
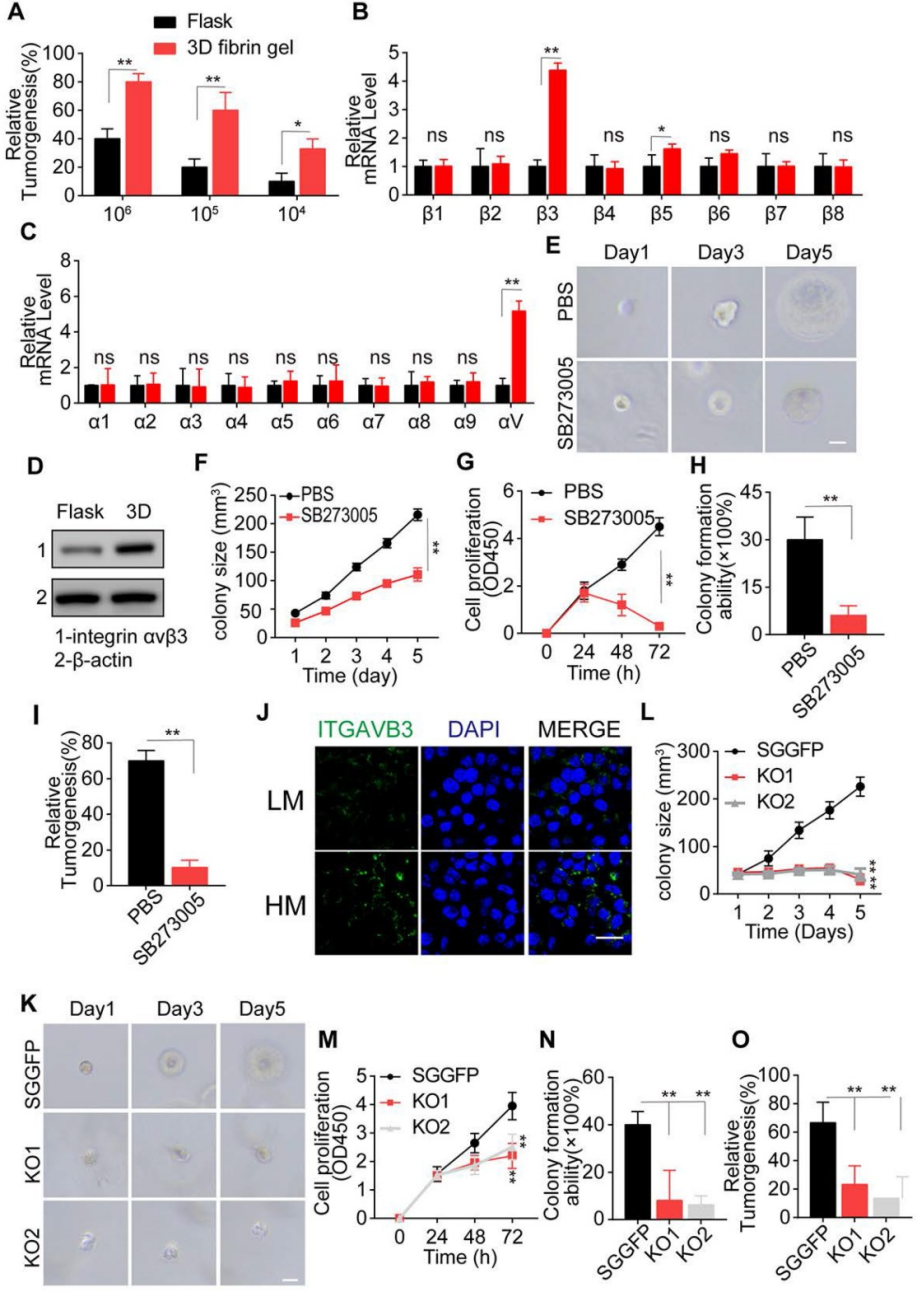
** 3D collagen I/FN gel induces tumor progression by integrin αvβ3** (A) Tumorigenesis of LN229 cells cultured in a flask or 3D fibrin gel in the NSG mouse (n=6). mRNA expression of integrin beta (B) or alpha (C) in LN229 cells cultured in a flask or 3D Collagen/FN gel for 48h. (D) Expression of integrin αvβ3 in LN229 cells cultured in a flask or 3D Collagen/FN gel for 48h. (E) Representative photographs of 3D collagen/FN cultured LN229 cells treated with PBS or SB273005 (5 nM). Scale bar represents 20 µm. (F) LN229 cells cultured in 3D collagen/FN gel were treated with PBS or SB273005(5 nM). Quantification of colony size is shown (n = 10). (G) Cell viability of 3D collagen/FN cultured LN229 cells treated with PBS or SB273005 (5 nM). (H) Colony formation analysis of 3D collagen/FN-cultured LN229 cells, treated with PBS or SB273005 (5 nM). (I) Tumorigenesis of 3D collagen/FN-cultured LN229 cells, treated with PBS or SB273005 (5 nM) (n=6). (J) Immunofluorescence staining of integrin αvβ3 in HM and LM glioma tissues. Scale bar represents 50 µm. (K) Representative photographs of 3D collagen/FN-cultured LN229-NC and LN229- ITGB3 KO cells. (L) Colony sizes of 3D collagen/FN-cultured LN229-NC and LN229- ITGB3 KO cells. (M) Cell proliferation of 3D collagen/FN-cultured LN229-NC and LN229- ITGB3 KO cells. (N) Colony formation of 3D collagen/FN cultured LN229-NC and LN229-ITGB3 KO cells. (O) Tumorigenesis of 3D collagen/FN cultured LN229-NC and LN229-ITGB3 KO cells. Mean ± SEM, n.s, no significant difference, *p < 0.05, **p < 0.01.

A compelling report has illustrated that 3D fibrin gel can induce stem-like phenotypes and promote tumor-repopulating cell growth through an integrin αvβ3-associated biomechanical signaling pathway 13. The results were duplicated in the LN229 and T98G glioma cells (Figure 2A and S2A). However, fibrin, a product of blood coagulation, is formed from fibrinogen by thrombin in the presence of calcium ions and does not exist in the tumor microenvironment 36. We speculated that type I collagen serves as the 3D skeleton for biomechanical signals, and fibrin analog FN uses membrane-spanning receptor protein integrin αvβ3 to transduce the pro-survival signal induced by biomechanical force in the tumor microenvironment, explaining the role of collagen and FN in glioma cell culture and tumor growth.

We first examined the expression of integrin β and α submits in LN229 and T98G cells and observed a significant up-regulation of integrin αv and β3 in 3D cultured tumor cells (Figure 2B, C, and S2B-C). Next, we examined the integrin αvβ3 protein expression in glioma cells cultured in 3D collagen/FN system for 3 days. As expected, elevated expression of integrin αvβ3 was observed in the 3D cultured tumor cells (Figure 2D and S2D). Blockade of integrin αvβ3 by SB273005, an integrin αvβ3 inhibitor, significantly suppressed cell proliferation and colony formation in the 3D collagen/FN system (Figure 2E, F and S2E-F). Similar results were observed in cell proliferation (Figure 2G and S2G), colony formation (Figure 2H and S2H) and tumorigenesis analysis (Figure 2I and S2I), indicating the crucial role of integrin αvβ3 in collagen/FN-induced tumor cell stemness. To eliminate the potential role of integrin β5 inhibition caused by SB273005, we knocked down integrin β3 in LN229 and T98G cells (Figure S2J) that suppressed the colony formation, cell proliferation, and tumorigenesis of glioma cells (Figure 2J-N and S2K-O). These observations were consistent with our previous results. Furthermore, integrin αvβ3 expression was also observed in malignant glioma tumor tissues from patients (Figure 2O). Together, these results suggested that collagen and FN facilitate glioma cell proliferation through integrin αvβ3 signals.

### 3D collagen I/FN gel promotes glioma growth via the integrin αvβ3/PI3K/AKT/SOX2 signaling pathway

**Figure 3:**
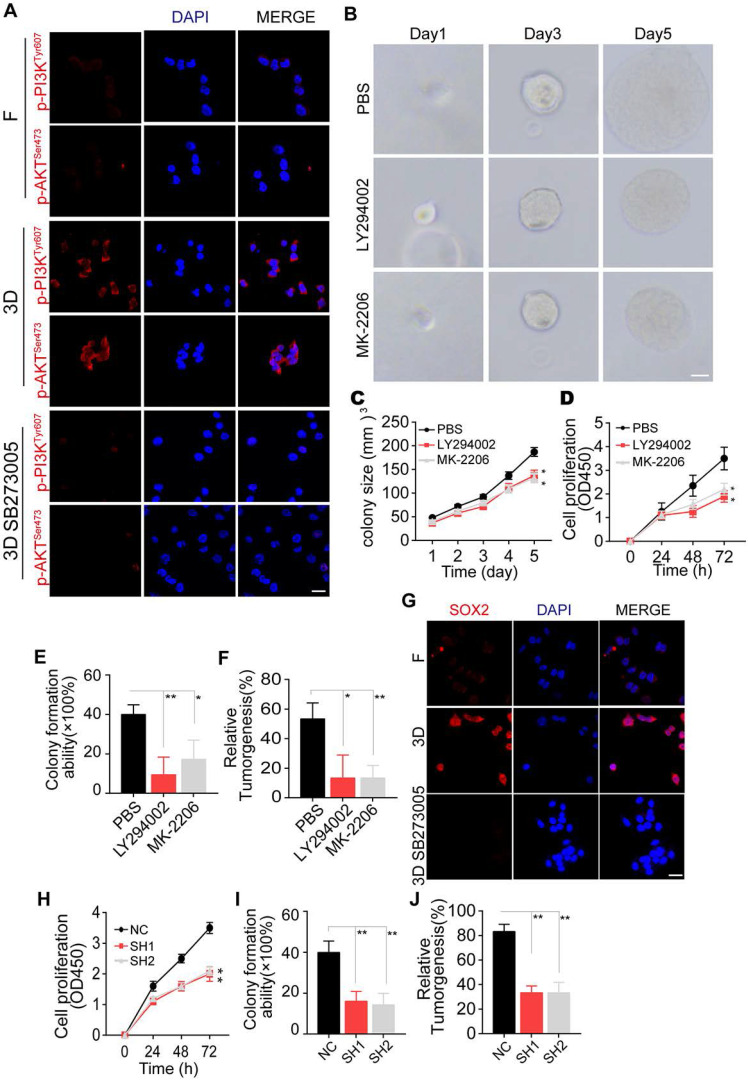
** 3D collagen I/FN gel promotes glioma growth via the integrin αvβ3/PI3K/AKT/SOX2 signaling pathway** (A) Immunofluorescence staining of p-PI3K^Tyr607^ and p-AKT^Ser473^ in LN229 cells treated with PBS or SB273005 (5 nM, 24 h), cultured in a flask or 3D collagen/FN environment. Scale bar represents 50 µm. (B) Representative photographs of 3D collagen/FN-cultured LN229 cells treated with PBS, LY294002 (10 µM, 24 h), or MK-2206 (0.5 µM, 24 h). Scale bar represents 20 µm. (C) Quantification of colony sizes in (B). (D) Proliferation of 3D collagen/FN-cultured LN229 cells treated with PBS, LY294002 (10 µM, 24 h), or MK-2206 (0.5 µM, 24 h). (E) Colony formation of 3D collagen/FN-cultured LN229 cells treated with PBS, LY294002 (10 µM, 24 h), or MK-2206 (0.5 µM, 24 h). (F) Tumorigenesis of 3D collagen/FN cultured LN229 cells treated with PBS, LY294002 (10 µM, 24 h), or MK-2206 (0.5 µM, 24 h) (n=6). (G) Immunofluorescence staining of SOX2 in LN229 cells cultured in a flask, 3D collagen/FN environment or 3D collagen/FN environment treated with SB273005 (5 nM). Scale bar represents 50 µm. (H) Cell viability of 3D collagen/FN-cultured LN229-NC cells or LN229 SOX2 KO cells. (I) Colony formation of 3D collagen/FN-cultured LN229-NC cells or LN229 SOX2 KO cells. (J) Tumorigenesis of 3D collagen/FN-cultured LN229-NC cells or LN229 SOX2 KO cells (n=6). Mean ± SEM, n.s, no significant difference, *p < 0.05, **p < 0.01.

It has been reported that ECM proteins, such as fibronectin and fibrinogen, could induce the PI3K/AKT pro-survival signaling pathway through integrin αvβ3 16, 30, 37. Herein, we detected PI3K and AKT expression in glioma cells cultured in 3D collagen/FN. Additionally, blockade of integrin αvβ3 in the 3D collagen/FN culture system suppressed p-PI3K and p-AKT expression, indicating that 3D collagen/FN culture induces PI3K/AKT activation *via* integrin αvβ3 (Figure 3A and Figure S3A). Consistent with this observation, inhibition of PI3K by LY294002 or AKT by MK-2206 suppressed glioma cell growth in the 3D collagen/FN system (Figure 3B-C and Figure S3B-C). The blockade of PI3K/AKT signal also inhibited the collagen/FN cultured glioma cell proliferation (Figure 3D and Figure S3D), colony formation (Figure 3E and Figure S3E), and tumorigenesis (Figure 3F and S3F), indicating the crucial role of PI3K/AKT signaling in glioma cells cultured in the 3D collagen/FN system.

The transcription factor SOX2, downstream of PI3K/AKT signaling, has been shown to regulate cell stemness and promote cell proliferation in several tumor types 38-40. We observed elevated expression of SOX2 in 3D collagen/FN cultured glioma cells, and blockade of PI3K/AKT suppressed this phenomenon (Figure 3G and Figure S3G). To further assess the role of SOX2 in glioma, we silenced SOX2 expression in LN229 and T98G cells by shRNA (Figure S3H) that efficiently suppressed the proliferation and colony formation capability induced by collagen/FN (Figure 3H-J and Figure S3I-K), indicating that SOX2 served as the downstream molecule of PI3K/AKT to induce glioma cell proliferation. Together, these results suggested that 3D collagen I/FN gel promotes glioma growth *via* the integrin αvβ3/PI3K/AKT/SOX2 signaling pathway.

### Integrin αvβ3/CDC42/F-actin/YAP/NUPR1/Nestin signaling pathway is activated in collagen/FN-cultured glioma cells

**Figure 4:**
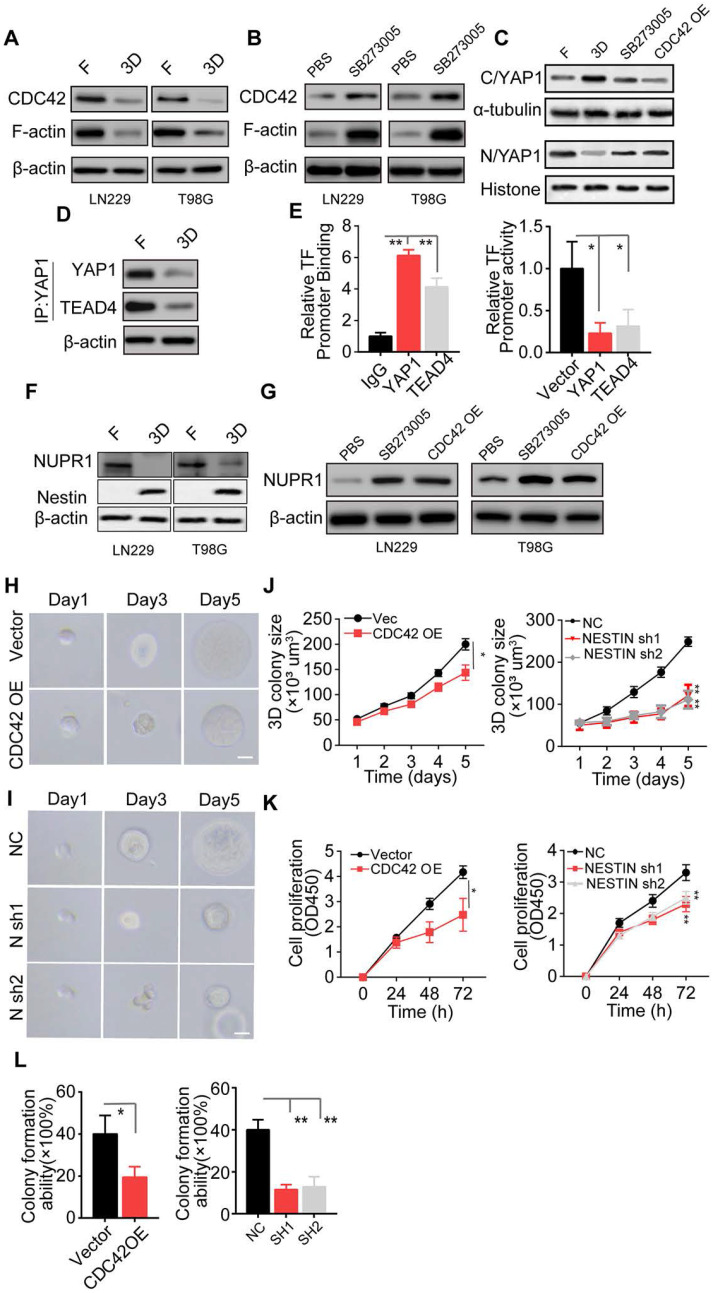
** The integrin αvβ3/CDC42/F-actin/YAP/NUPR1/Nestin signaling pathway is activated in collagen/FN-cultured glioma cell** (A) Expression of CDC42, F-actin, and β-actin in LN229 cells and T98G cells cultured in a flask or 3D Collagen/FN gel. (B) Expression of CDC42, F-actin, and β-actin in LN229 cells and T98G cells cultured in 3D Collagen/FN gel treated with PBS or SB273005 (5 nM, 24). (C) Cell fraction of cytosol (C) and nucleus (N) was analyzed by Western blotting. Expression of YAP1 in LN229 cells or LN229 CDC42 OE cells cultured in a flask or 3D Collagen/FN gel and treated with PBS or SB273005 (5 nM, 24 h). (D) Western blotting analysis of proteins immunoprecipitated (IP) with anti-YAP1 from LN229 cells cultured in a flask or 3D Collagen/FN gel for 48 h. (E) ChIP analysis of YAP1 or TEAD4 binding to the NUPR1 promoter in LN229 cells. YAP1 and TEAD4 increased the luciferase activity of the NUPR1 promoter in LN229 cells. (F) Expression of NUPR1 and Nestin in LN229 cells and T98G cells cultured in a flask or 3D Collagen/FN gel. (G) Expression of NUPR1in LN229 and T98G cells cultured in 3D Collagen/FN gel, treated with PBS or SB273005 (5 nM, 24 h) and transfected with CDC42 OE lentivirus. (H) Representative photographs of LN229-NC cells and LN229-CDC42 OE cells cultured in 3D Collagen/FN gel. Scale bar represents 20 µm (I) Representative photographs of LN229-NC cells and LN229-Nestin shRNA cultured in 3D Collagen/FN gel. Scale bar represents 20 µm (J) Quantification of colony sizes in (H) and (I) (K) Proliferation of 3D collagen/FN pre-cultured LN229-vec cells, LN229-CDC42 OE cells, LN229-NC cells, and LN229-Nestin shRNA cells (L) Colony formation of 3D collagen/FN pre-cultured LN229-vec cells, LN229-CDC42 OE cells, LN229-NC cells, and LN229-Nestin shRNA cells. Mean ± SEM, n.s, no significant difference, *p < 0.05, **p < 0.01.

Our results showed that the PI3K/AKT/SOX2 axis is critical in glioma cell proliferation induced by collagen/FN. However, SOX2 may not be the only critical factor in glioma progression. Previous data showed that blockade of PI3K/AKT/SOX2 did not completely suppress glioma cell proliferation cultured in the 3D collagen/FN system and a high percentage of cells still displayed the proliferative phenotype (Figure 3D and S3D), suggesting that growth regulation of glioma by the collagen/FN system is complex. As a key cytoskeleton regulator, CDC42 is known to regulate the actin cytoskeleton and promote filopodia formation. CDC42, which is downstream of integrins, has been demonstrated to transduce biodynamic signals 41, 42. Here, the downregulation of CDC42 and cytoskeletal protein F-actin was observed in 3D collagen/FN cultured glioma cells, whereas blockade of integrin αvβ3 reversed the phenomenon (Figure 4A-B). Yes-associated protein, YAP-1, a mediator of Hippo signaling, is known to sense cytoskeletal tension and mediate cellular biomechanical responses 43. We observed reduced nuclear but elevated cytoplasmic YAP-1 in the 3D collagen/FN-cultured glioma cells, whereas integrin αvβ3 suppression or CDC42 overexpression significantly increased the YAP-1 expression in the nucleus (Figure 4C, Figure S4A and B). It is worth noting that YAP-1 does not stimulate gene transcription unless it interacts with co‐activator proteins such as TEAD (TEA/ATTS domain) transcription factors or TAZ (transcriptional co‐activator with PDZ‐binding motif) 44. Mass spectrometry analysis showed that TEAD4 may be the main binding partner for YAP1 as a co‐activator protein in glioma (Figure S4C-D). The Co-IP assay confirmed direct interaction of YAP1 with TEAD4 in glioma cells cultured in a flask (Figure 4E and S4D). These results indicated that 3D collagen/FN culture mediates activation of the integrin αvβ3/CDC42/F-actin/YAP-1 signaling.

NUPR1, a DNA-binding protein associated with cell cycle regulation and apoptosis, has been shown to be involved in tumor progression 45. Nestin, a downstream transcription factor of NUPR1, is associated with glioma development and has been demonstrated to be negatively regulated by NUPR1 46. Interestingly, the binding affinity between YAP1-TEAD4 was decreased in 3D collagen/FN culture, which may be attributed to reduced YAP-1 nuclear location. Our ChIP and dual-luciferase analyses found that YAP-1 and TEAD4 were enriched in the promoter sites of NUPR1 and enhanced its expression. (Figure 4E and Figure S4F-G), indicating that intranuclear YAP-1 and TEAD4 might induce NUPR1 activation and negatively regulate Nestin expression. As expected, reduced NUPR1 and increased Nestin expression were observed in 3D collagen/FN cultured glioma cells, whereas blockade of integrin αvβ3 or CDC42 overexpression reversed the phenomenon (Figure 4F-G). These results indicated that 3D collagen/FN culture induced αvβ3/CDC42/F-actin/YAP-1/NUPR1/Nestin signaling pathway activation. To further demonstrate the role of this pathway in glioma progression, we established Nestin knockdown LN229/T98G cells (Figure S4H and I). Consistent with our previous results, CDC42 overexpression or Nestin knockdown suppressed 3D colony growth (Figure 4H-J and Figure S4J-L), proliferation (Figure 4K and S4M), and colony formation (Figure 4L and S4N). Together, these results suggested that the integrin αvβ3/CDC42/F-actin/YAP-1/NUPR1/Nestin signaling pathway was activated in 3D collagen/FN cultured glioma cells and induced glioma cell proliferation.

### Blockade of SOX2 and Nestin signals efficiently suppresses glioma cell proliferation

**Figure 5:**
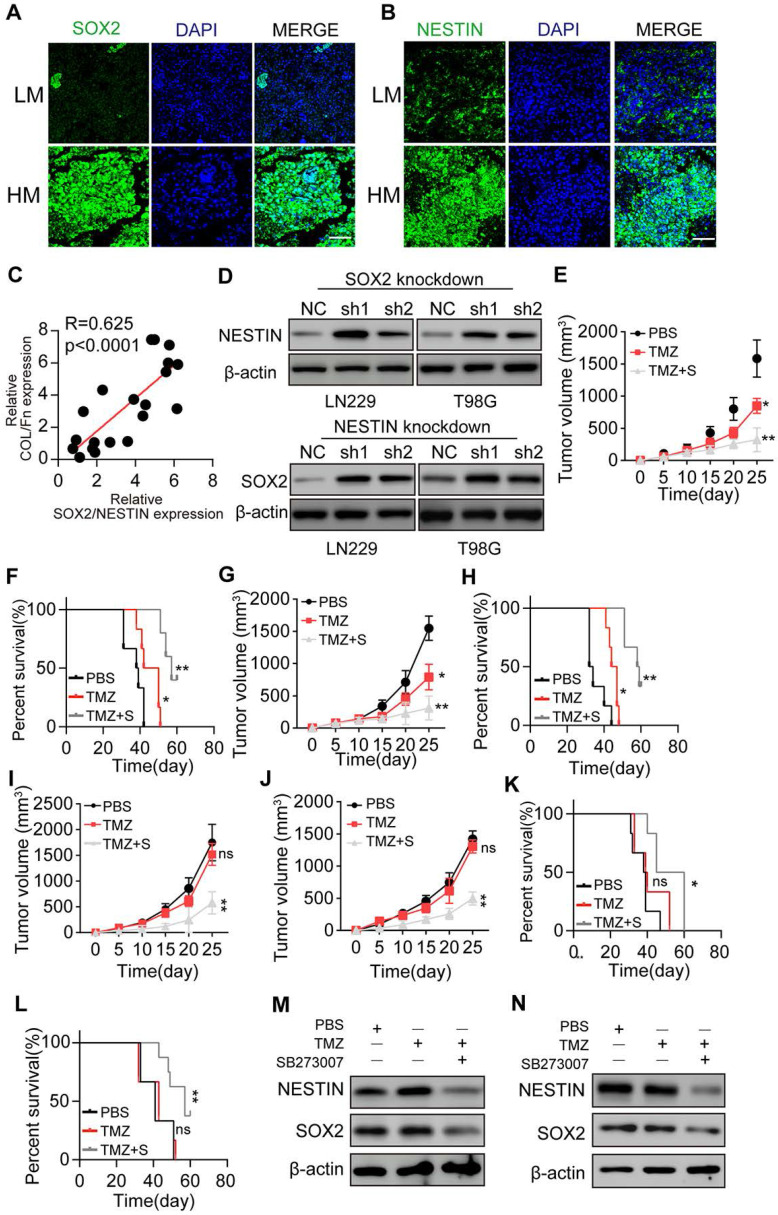
** Blockade of SOX2 and Nestin signals efficiently suppresses glioma cell proliferation.** (A) Immunofluorescence staining of SOX2 in HM or LM glioma tissues from patients. Scale bar represents 200 µm (B) Immunofluorescence staining of Nestin in HM or LM glioma tissues from patients. Scale bar represents 200 µm (C) Correlation analysis between the SOX2/Nestin high expression group and Collagen/FN high expression group in glioma patients. (D) Expression of Nestin and SOX2 in LN229-NC cells, LN229-SOX2 KO cells, T98G-NC cells, and T98G-SOX2 KO or T98G-NESTIN KO cells, respectively. Tumor volumes in animals implanted with LN229 cells (E) or T98G cells (F) treated with PBS, TMZ (10 mg/kg), or TMZ (10 mg/kg) combined with SB273005 (30 mg/kg) by oral administration. Survival time of animals implanted with either LN229 cells (G) or T98G cells (H) treated with either PBS, TMZ (10 mg/kg), or TMZ (10 mg/kg) combined with SB273005(30 mg/kg) by oral administration. Tumor volume of animals implanted with either LN229 cells (I) or T98G cells (J) cultured in 3D Collagen/FN environment for 3 days, treated with either PBS, TMZ (10 mg/kg) and TMZ (10 mg/kg) combined with SB273005 (30 mg/kg) by oral administration. For collagen and FN environment *in vivo*, Collagen /FN gel was injected into the tumor sites on day 7 and 14 (K) Survival time of animals in (I). (L) Survival time of animals in (J). Expression of SOX2 or Nestin in LN229 tumor tissues (M) or in T98G tumor tissues (N) treated with either DMSO, TMZ (10 mg/kg) and TMZ (10 mg/kg) combined with SB273005 (30 mg/kg) by oral administration. Mean ± SEM, n.s, no significant difference, *p < 0.05, **p < 0.01.

Our *in vitro* results demonstrated that 3D collagen/FN culture could induce the PI3K/AKT and CDC42/YAP/NUPR1 signal activation in glioma cells, resulting in growth factors SOX2/Nestin up-regulation. To verify our data *in vivo*, we examined glioma tissues from patients and found elevated SOX2 and Nestin expression in high-grade malignant glioma tissues (Figure 5A and B). Significantly, a high correlation was observed between the collagen+/FN+ and SOX2+/Nestin+ groups (Figure 5C), consistent with our *in vitro* results. Also, PI3K/AKT inhibitors or Nestin suppression did not eliminate the colony formation or suppress glioma cell proliferation. We examined the SOX2 and Nestin expression in SOX2 or Nestin knockdown glioma cells cultured in the 3D collagen/FN system. The expression of Nestin was upregulated in SOX2 knockdown glioma cells (Figure 5D), sand, conversely, SOX2 was upregulated in Nestin knockdown glioma cells. These results indicated that glioma cell proliferation could be regulated by the pro-survival signaling network or signaling pathway inhibitor treatment, such as PI3K/AKT inhibitors.

We used LN229 or T98G cells to establish a subcutaneous glioma mouse model for anticancer treatment analysis. SB273005, in combination with TMZ treatment, significantly suppressed the LN229 glioma growth and prolonged the survival time of tumor-bearing mice (Figure 5E-F). Similar results were observed in T98G tumor-bearing mice (Figure 5G-H). To simulate clinical malignant glioma, we used 3D collagen/FN cultured LN229 cells to establish a glioma mouse model. Collagen and FN were injected into the tumor sites on days 7 and 14 to induce high-grade malignant glioma. Subsequently, the combination of SB273005 and TMZ was used by oral administration for glioma treatment. The single-agent TMZ treatment revealed a poor outcome, which might be due to the drug resistance development caused by sustained tumor growth 47. However, the combination of SB273005 and TMZ efficiently suppressed tumor growth and prolonged survival time (Figure 5I-L). Western blotting of tumor tissues demonstrated efficient SOX2/Nestin suppression in SB273005 or SB273005/TMZ groups compared to the control or TMZ group (Figure 5M-N). Together, those results indicated that suppressing SOX2 and Nestin signaling by the integrin αvβ3 inhibitor efficiently improved the anticancer effects of the conventional chemotherapeutic agent, providing an innovative approach in glioma therapy.

## Discussion

Increasing evidence has implicated cancer cells with stem-like phenotype in eliciting frequent tumor recurrence in patients who temporarily benefit from surgical resection and/or chemotherapy 11, 48-50. However, the concept of cancer stem cells remains controversial with the query of whether these are intrinsic entities or plastic cells driven by microenvironmental cues 13, 21. Our study described a novel *in vitro* 3D cell culture model to induce glioma cells' aggressive proliferation and tumorigenesis, supporting the concept of bidirectional interconversion between differentiated cells and stem cells in the specific tumor microenvironment.

Little is known about the bidirectional interconversion between cancer stem cells and bulk tumor cells. Compelling reports have shown that several extracellular matrix components strongly correlate with cancer development 51, 52. For example, the elevated fibrinogen in the tumor site increased the survival and proliferative potential of colorectal cancer cells 53. The collagen complex was reported to promote ovarian cancer metastasis and accelerate drug-resistance development in breast carcinoma 54, 55. Recent studies also demonstrated the establishment of *in vitro* 3D fibrin gel culture systems to promote cells with stem-like phenotypes in breast cancer 13. Liu and colleagues reported the advantages of promoting the selection and growth of tumorigenic cells in a 3D soft fibrin gel system, resulting in elevated stem cell-like phenotypes in multifarious tumor cells 13, 16. However, the promotion of stem-like cancer cells failed to be duplicated in most cancer cell lines 56-58. Also, the high concentration of fibrin (1 mg/mL) used in the 3D fibrin model does not exist *in vivo*, and the low distribution of fibrin does not support tumor stemness modification in the tumor microenvironment 36, 59.

We used type I collagen to formulate the 3D inner structure and FN to replace fibrin for binding to integrin αvβ3 and transducing the biodynamic signal from the extracellular 3D collagen constitution. Our results showed that 3D collagen/FN culture efficiently facilitated glioma stem cell-like invasive and tumorigenic phenotype, indicating synergistic roles of collagen and FN in the extracellular matrix. We also confirmed the high expression of collagen and FN in malignant glioma tissues promoting the stem-like phenotype of glioma cells. Furthermore, we observed PI3K/AKT activation through the cell surface receptor integrin αvβ3, a crucial pro-survival signal involved in the sustained growth of various cancers. Previous reports have demonstrated that type I collagen could elicit sustained tumor growth by stimulating PI3K/AKT signaling 33, 60 downstream of the integrin signaling 61. In contrast with previous findings, we showed that glioma cells cultured in 3D collagen/FN reacquired stem-like characteristics through the integrin αvβ3/PI3K/AKT cascade, which further induced the downstream transcription factor SOX2 activation. Additionally, PI3K/AKT inhibitors failed to completely suppress glioma cell proliferation in the 3D collagen/FN structure. We, therefore, investigated the underlying molecular mechanisms of glioma cells stem-like phenotype with biodynamic-associated pro-survival signals induced by integrins. It has previously been shown that the integrin-FAK-CDC42 signaling axis could modulate actin cytoskeletal distribution and YAP phosphorylation, resulting in altered YAP localization and eventually inducing cell proliferation through downstream transcriptional factors 62. We identified the integrin αvβ3/CDC42/F-actin/YAP-1/Nupr1/Nestin signaling pathway, which was independent of the PI3K/AKT signaling in 3D collagen/FN cultured glioma cells. We further demonstrated the sustained tumor growth of glioma when PI3K/AKT signaling was suppressed, providing an explanation for the clinical failure of single small molecule inhibitor.

Given the crucial role of integrin αvβ3 in collagen/FN-induced tumor progression in glioma, it might be feasible to eradicate tumor cells with stem-like phenotypes by targeting and inhibiting integrin αvβ3 and suppress glioma development. Therefore, a better understanding of integrin-induced mechanisms in tumor progression is necessary for the application of integrin inhibitors 63, 64. The integrin αvβ3 inhibitor cilengitide failed to improve carcinoma patients' outcome in a Phase III clinical trial. implying that the tumorigenic potential can be maintained despite a low proliferative rate in the absence of integrin signaling 65, 66. The tumor cells could rapidly reacquire sustained proliferative characteristics upon terminating integrin inhibitor administration 67.

Thus, targeting glioma cells by co-delivering integrin αvβ3 inhibitor SB273005 with the chemotherapeutic TMZ might be advantageous to suppress αvβ3-positive tumor cells induced by collagen/FN as well as the integrin αvβ3 negative tumor cells with a low proliferative rate. More importantly, oral SB273005 effectively crosses the blood-brain barrier. A combination of SB273005 and TMZ, when administered orally, an advantageous delivery route 68, efficiently suppressed the activation of pro-tumor transcription factors SOX2 and Nestin expression, leading to superior tumor suppression and prolonged overall survival time in orthotopic glioma models 69, 70.

To address the limitations of existing glioma treatment strategies, we further illustrated the crucial roles of type I collagen/FN in regulating glioma progression. First, we confirmed the correlation of type I collagen and FN in glioma cell stemness modification, demonstrating that in the 3D system, collagen could induce the biodynamic signal *via* FN interaction with cell surface receptor integrin αvβ3 in glioma cells. The elevated expression of collagen/FN in high-grade gliomas also underscored the relevance of ECM in tumor development. Based on these findings, we developed a novel 3D collagen/FN culture system as an optional model to simulate tumor microenvironment *in vitro* for culturing cancer stem cells. Second, we elucidated the underlying mechanism of tumor progression induced by the collagen/FN complex. We provided evidence that collagen/FN complex regulates glioma stemness *via* an integrin αvβ3-activated PI3K/AKT/SOX2 and CDC42/F-actin/YAP-1/Nupr1/Nestin signaling network. And third, the combination of integrin inhibitor SB273005 and chemotherapy revealed superior tumor suppression and prolonged overall survival in the glioma tumor model, providing an excellent clinical strategy. Finally, the expression level of type I collagen and FN in tumor tissues might serve as potential biomarkers for monitoring tumor progression during glioma treatment.

## Conclusions

Our study provided evidence suggesting that collagen and FN collaborate to facilitate proliferation and tumorigenesis of glioma cells through the PI3K/AKT/SOX2 and CDC42/F-actin/YAP-1/Nupr1/Nestin pro-survival signaling networks. Inhibition of the upstream integrin αvβ3 signal offers a promising clinical exploitable therapeutic approach to retard tumor proliferative signals, strengthen chemotherapy response, and efficiently suppress glioma progression, providing a potentially superior treatment strategy.

## Supplementary Material

Supplementary figures.

## Abbreviations

CSCs: Cancer Stem Cell
DMEM: Dulbecco's Modified Eagle's Medium
ECM: extracellular matrix
KDa: kilo Dalton
FN: fibronectin
ShRNA: short hairpin RNA
3D: 3-dimension
2D: 2-dimension
NC: negative control
YAP1: Yes1 associated transcriptional regulator
CDC42: cell division cycle 42
AKT: AKT serine/threonine kinase 1
PI3K: phosphatidylinositol 3-kinase
NUPR1: nuclear protein 1 transcriptional regulator
F: Flask
PBS: phosphate-buffered saline
