# Supplementary material for: Remodeling cancer stemness by collagen/fibronectin via the AKT and CDC42 signaling pathway crosstalk in glioma
Source: Theranostics. 2021 Jan 1;11(4):1991–2005. doi: 10.7150/thno.50613 (PMC7778591; doi:10.7150/thno.50613)
Supplement: Supplementary file 1 — Supplementary figures. [file thnov11p1991s1.pdf]

**Supplementary information**

Figure S1

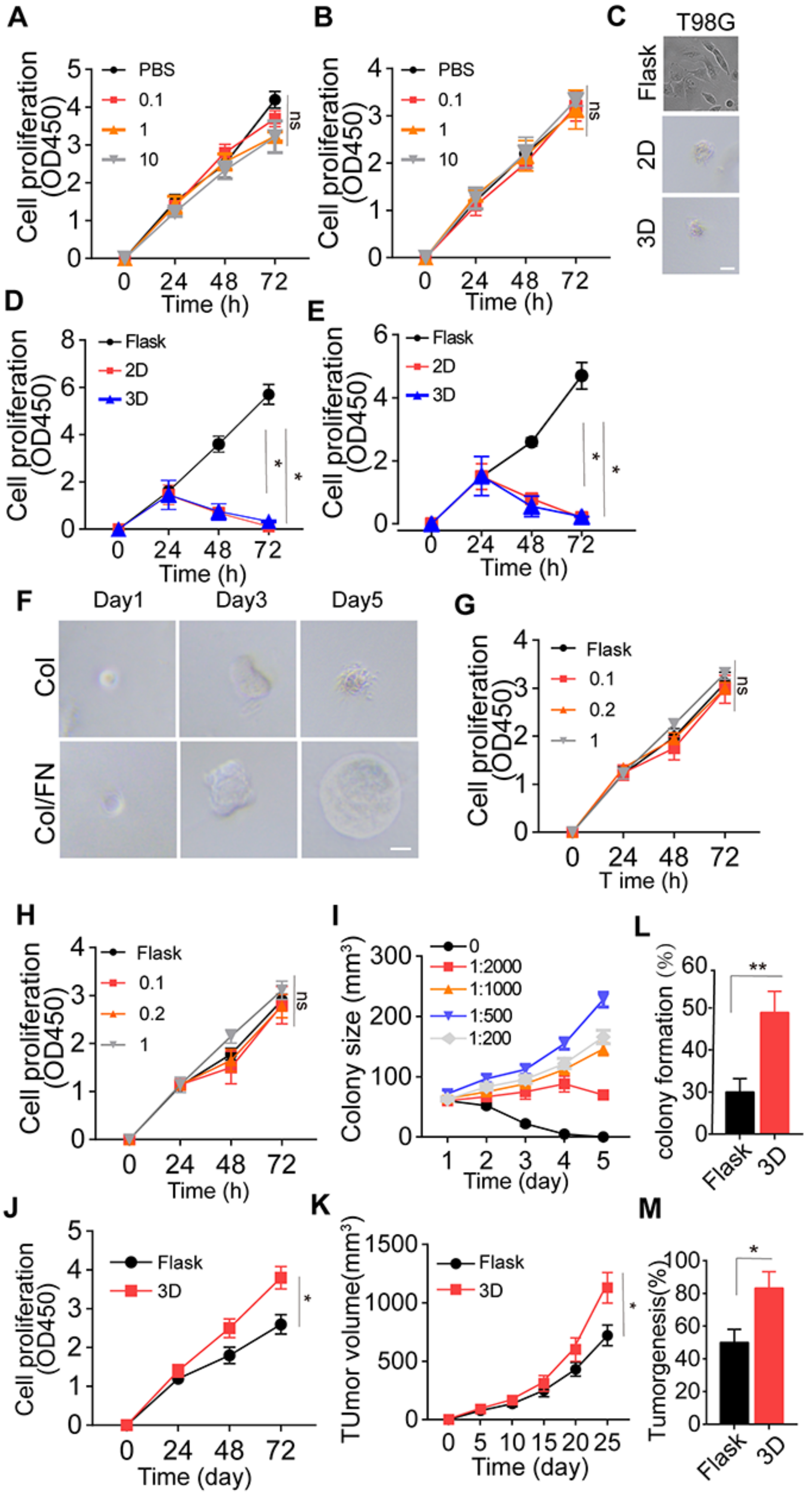

(A) The cell viability of LN229 cells treated with type I collagen (0.1 -10μg/mL) by CCK-8 assay. (B) The cell viability of T98G cells treated with type I collagen (0.1-10μg/mL) by CCK-8 assay. (C)The representative microscope picture of T98G cells cultured in flask, 2D collagen environment or 3D collagen environment for 72 hours. Scale bar represents 20 μm. (D) The cell viability of LN229 cells cultured in flask, 2D collagen environment and 3D collagen environment. (E) The cell viability of T98G cells cultured in flask, 2D collagen environment and 3D collagen environment. (F) The representative microscope picture of T98G cells cultured in 3D collagen environment with PBS or FN (FN: collagen, 1:500). Scale bar represents 20 μm. (G) The cell viability of LN229 cells treated with FN (0.1 -1μg/mL) using the CCK-8 assay. (H) The cell viability of T98G cells treated with FN (0.1-1μg/mL) using the CCK-8 assay. (I) T98G cells cultured in 3D collagen environment which was added to FN. Quantification of colony size is shown (n = 10). (J) The cell viability of T98G cells cultured in flask or 3D Collagen/FN environment using the CCK-8 assay. (K) Tumor volume of xenografts in NSG mice (n = 6) by $1\times10^6$ T98G cells cultured in flask or 3D Collagen/FN environment. (L) The colony formation of T98G cells cultured in flask or 3D Collagen/FN environment. (M) The tumorigenesis of T98G cells cultured in flask or 3D Collagen/FN environment (n = 6). Mean ± SEM, n.s, no significant difference, *$p < 0.05$, **$p < 0.01$.

Figure S2

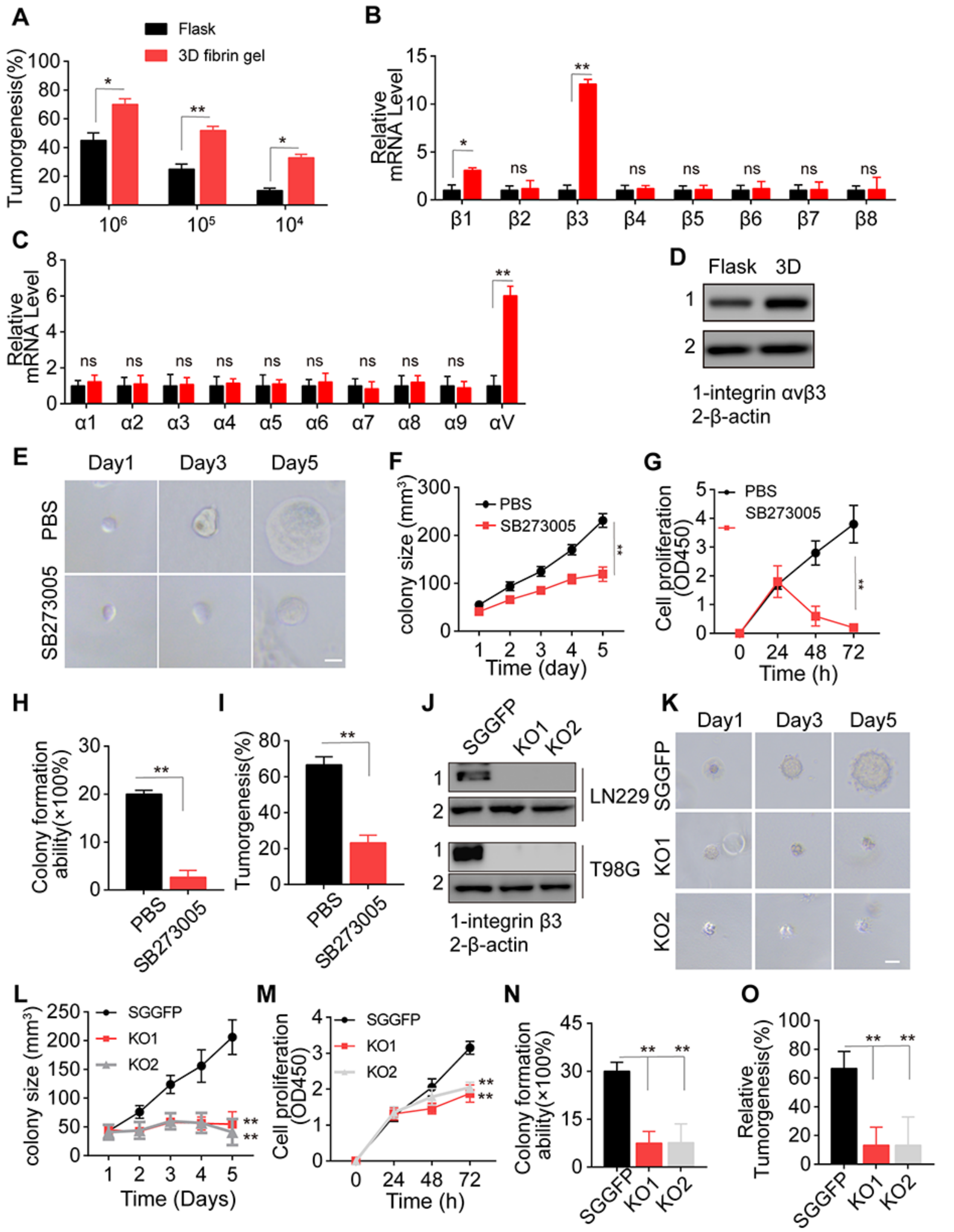


(A) The tumorigenesis of T98G cells cultured in flask or 3D fibrin gel in NSG mouse (n =6). The mRNA expression of integrin beta(B) or alpha (C) in T98G cells cultured in flask or 3D Collagen/FN gel for 48h. (D) The expression of integrin αvβ3 in T98G cells cultured in flask or 3D Collagen/FN gel. (E) The representative microscope pictures of 3D collagen/FN cultured T98G cells treated with PBS or SB273005 (5 nM). The scale bar represents 20 µm. (F) T98G cells cultured in 3D collagen/FN environment were treated with PBS or SB273005(5 nM). Quantification of colony size is shown (n = 10). (G) The cell viability of 3D collagen/FN cultured T98G cells

were treated with PBS or SB273005 (5 nM). (H) The colony formation analysis of 3D collagen/FN cultured T98G cells, which were treated with PBS or SB273005 (5 nM). (I) The tumorigenesis of 3D collagen/FN cultured T98G cells, which were treated with PBS or SB273005 (5 nM) (n =6). (J) The western bolting of ITGB3 in LN299-NC, LN229 ITGB3 KO, T98G-NC cells or T98G ITGB3 KO cells. (K) The representative microscope picture of T98G cells cultured in 3D collagen/FN cultured T98G-NC cells, T98G- ITGB3 KO cells. (L) The colony sizes of T98G cells cultured in 3D collagen/FN cultured T98G-NC cells, T98G- ITGB3 KO cells. (M) The cell proliferation of 3D collagen/FN cultured T98G-NC cells, T98G- ITGB3 KO cells. (N) The colony formation of 3D collagen/FN cultured T98G-NC cells, T98G-ITGB3 KO cells. (O) The tumorigenesis of 3D collagen/FN cultured T98G-NC cells, T98G-ITGB3 KO cells. Mean ± SEM, n.s, no significant difference, *$p < 0.05$, **$p < 0.01$.

s

Figure S3

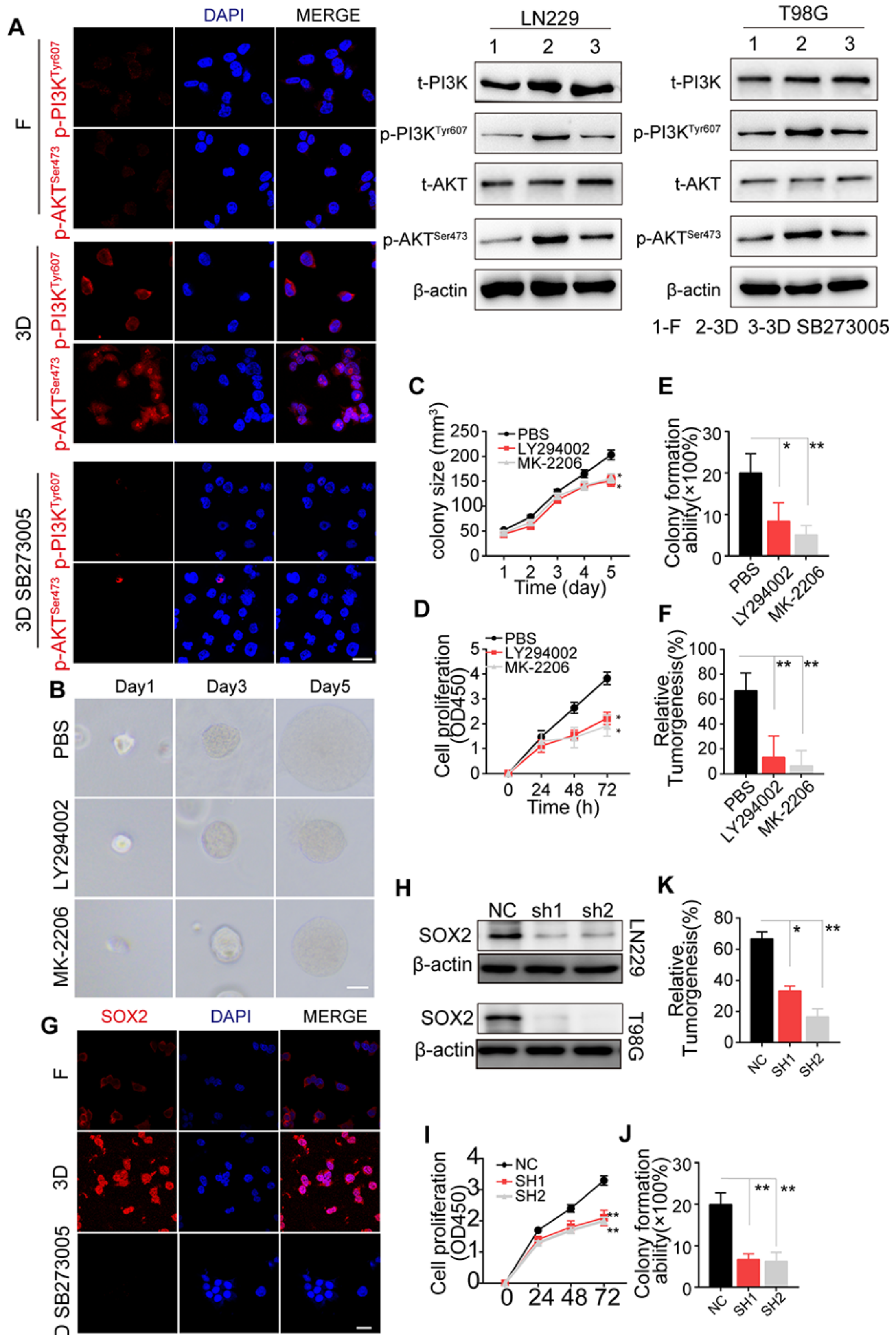


(A) The immunofluorescence staining of p-PI3K$^{Tyr607}$, p-AKT$^{Ser473}$ in T98G cells treated with PBS or SB273005(5 nM, 24 h), which cultured in flask or 3D collagen/FN environment. Scale bar represents 50 μm. The western blotting of p-PI3K$^{Tyr607}$, p-AKT$^{Ser473}$, t-AKT and t-PI3K in in LN229 or T98G cells treated with

PBS or SB273005 (5 nM, 24 h), which cultured in flask or 3D collagen/FN environment. (B) The representative microscope picture of 3D collagen/FN cultured T98G cells treated with PBS, LY294002(10 μM, 24 h) and MK-2206 (0.5 μM, 24 h). Scale bar represents 20 μm. (C) Quantification of colony sizes in (B) is shown. (D) The cell proliferation of 3D collagen/FN cultured T98G cells treated with PBS, LY294002 (10 μM, 24 h) and MK-2206 (0.5 μM, 24 h). (E) The colony formation of 3D collagen/FN cultured T98G cells treated with PBS, LY294002 (10 μM, 24 h) and MK-2206 (0.5 μM, 24 h). (F) The tumorigenesis of 3D collagen/FN cultured T98G cells treated with PBS, LY294002(10 μM, 24 h) and MK-2206 (0.5 μM, 24 h) (n =6). (G) The immunofluorescence staining of SOX2 in T98G cells cultured in flask, 3D collagen/FN environment or 3D collagen/FN environment treated with SB273005 (5 nM). Scale bar represents 50 μm. (H) The western bolting of SOX2 in LN299-NC, LN229 SOX2 KO, T98G-NC cells or T98G SOX2 KO cells. (I) The cell viability of 3D collagen/FN cultured T98G-NC cells or T98G SOX2 KO cells. (J) The colony formation of 3D collagen/FN cultured T98G-NC cells or T98G SOX2 KO cells. (K) The tumorigenesis of 3D collagen/FN cultured T98G-NC cells or T98G SOX2 KO cells (n =6). Mean ± SEM, n.s, no significant difference, *$p < 0.05$, **$p < 0.01$.

Figure S4

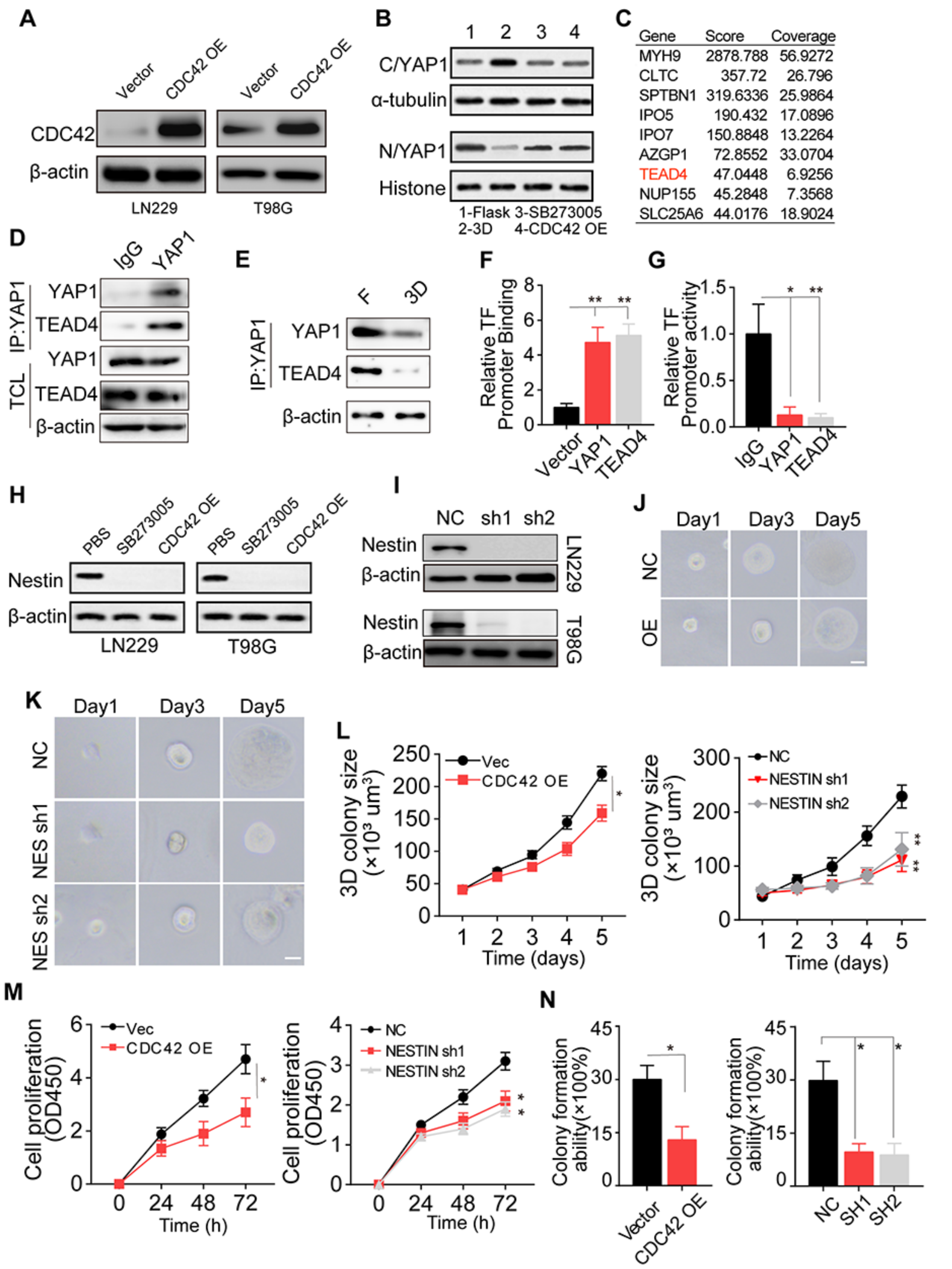


(A) The western blotting of CDC42 in LN229-NC, LN229 CDC42 OE, T98G NC and T98G CDC42 OE. (B) Cell fraction of cytosol (C) and nucleus (N) was analyzed by western blot. The expression of YAP1 in T98G cells or T98G CDC42 OE cells cultured in flask or 3D Collagen/FN gel, which were treated with PBS or SB273005(5 nM, 24 h). (C) The LN229 cells were lysed and immunoprecipitated with anti-YAP1or IgG antibody. The proteins immunoprecipitated were run on a SDS-PAGE gel and processed for mass spectrometry. (D) The western blotting analysis of total LN229 cell lysates (TCL, bottom) and proteins immunoprecipitated

(IP) with anti-YAP1 or control immunoglobulin G (IgG)(top) cells. (E) The western blotting analysis of proteins immunoprecipitated (IP) with anti-YAP1 from T98G cells cultured in flask or 3D Collagen/FN gel. (F) ChIP analysis of YAP1 or TEAD4 binding to the NUPR1 promoter in T98G. (G) YAP1 or TEAD4 increased the luciferase activity of the NUPR1 promoter in T98G cells. (H) The expression of Nestin in LN229 and T98G cells cultured in 3D Collagen/FN gel, which were treated with PBS or SB273005 (5 nM, 24 h) and CDC42 overexpression. (I) The expression of Nestin in LN229-NC cells, LN229-Nestin shRNA cells, T98G-NC cells and T98G-Nestin shRNA cells. (J) The representative microscope pictures of T98G-NC cells and T98G-CDC42 OE cultured in 3D Collagen/FN gel. Scale bar represents 20 μm. (K) The representative microscope pictures of T98G-NC cells and T98G-Nestin shRNA cultured in 3D Collagen/FN gel. (L) Quantification of colony size (J) and (K) is shown. (M) The proliferation of 3D collagen/FN cultured T98G-vec cells, T98G-CDC42 OE cells, T98G-NC cells and T98G-Nestin shRNA cells. (N) The colony formation of 3D collagen/FN cultured T98G-vec cells, T98G-CDC42 OE cells, T98G-NC cells and T98G-Nestin shRNA cells. Mean ± SEM, n.s no significant difference, *$p < 0.05$, **$p < 0.01$.
